# Molecular Dynamics-Guided Triage of Approved Drugs Against *Escherichia coli* Resistance Targets Nominates Capmatinib, Metergoline and Nilotinib as Candidates for Experimental Testing

**DOI:** 10.3390/ijms27209017

**Published:** 2026-10-12

**Authors:** Mena Abdelsayed, Yassir Boulaamane

**Affiliations:** 1 Lankenau Institute for Medical Research, Philadelphia, PA 19096, USA; 2 Laboratory of Innovative Technologies, National School of Applied Sciences of Tangier, Abdelmalek Essaadi University, Tetouan 93000, Morocco

**Keywords:** antimicrobial resistance, drug repurposing, molecular docking, molecular dynamics, capmatinib, dihydrofolate reductase, hydrogen bonding, scoring-function bias, FabI, LpxC

## Abstract

Virtual screening ranks compounds by scoring functions that correlate with molecular size, and hit lists are rarely benchmarked against compounds of established activity. We screened 2070 approved drugs against four *Escherichia coli* targets selected by pocket-druggability triage (FabI, LpxC, DHFR and GyrB), with each docking protocol validated by redocking its native ligand (RMSD 0.50–0.69 Å). Per target, the two top-scoring compounds and a reference inhibitor were simulated for 100 ns in triplicate (12 complexes, 3.6 μs). Docking did not recover the references: trimethoprim ranked 762/2068 and novobiocin 576/2069, while top-ranked compounds were large, promiscuous drugs recurring across unrelated targets. Pose triage failed, too: all twelve complexes retained 43–87% of initial contacts, contact-loss time separated targets, not compounds, and MM-GBSA favoured the reference at one target of four. Persistent hydrogen bonding separated the reference from the hits at three of four targets, by 2–9-fold, although the GyrB result rests largely on one replicate. Benchmarking each hit against its reference nominates three candidates for experimental testing: capmatinib at DHFR, metergoline at FabI and nilotinib at LpxC. Capmatinib ranks highest on these computational criteria, with the highest pocket retention (87%) and an MM-GBSA energy with no detectable difference from trimethoprim’s at n = 3 (absence of evidence for a difference, not evidence of equivalence). In this small panel, carrying a known active through the same pipeline was therefore more informative than docking score, pose stability or end-point rescoring. The nominations are hypotheses: no experimental validation was performed, and generality would require multiple actives and property-matched decoys per target.

## 1. Introduction

Antimicrobial resistance was associated with an estimated 4.95 million deaths in 2019, of which 1.27 million were directly attributable [1], and the most recent Global Research on Antimicrobial Resistance analysis forecasts that the burden will continue to rise through to 2050 [2]. The antibacterial pipeline has not kept pace, and few mechanistically novel agents against Gram-negative pathogens have reached the clinic in decades. Repurposing approved drugs is an attractive response, because pharmacokinetics, toxicology, formulation and manufacturing are already established, which removes much of the attrition that dominates early development [3]. In the antibacterial setting specifically, repurposing has yielded several credible candidates and has the further advantage that a compound’s human safety profile is known before any antibacterial claim is made [4].

The computational form of this strategy is to dock an approved-drug library against a bacterial target, rank by score, and advance the top of the list. That workflow rests on two assumptions that are seldom tested within the same study: that the scoring function ranks true actives above inactives, and that the target site can bind a drug-like ligand at all.

Neither assumption is safe, and both fail in identifiable ways. Docking programmes reproduce crystallographic poses considerably more reliably than they rank affinities, an asymmetry documented across many programmes and targets [5,6]. Vina-class empirical functions in particular are dominated by buried non-polar contact area, and therefore systematically reward large, lipophilic molecules irrespective of whether the buried surface corresponds to specific recognition [7,8]. Size-normalised measures such as ligand efficiency and the related drug-efficiency indices were introduced precisely to counter this distortion [9,10], and property-matched decoy sets were built so that enrichment could be measured without it [11]. The practical consequence for an approved-drug library is that the same handful of large, promiscuous compounds surfaces at the top of mechanistically unrelated target lists, a pattern familiar from screening-library curation more broadly [12].

The second assumption concerns the target rather than the compound. Many attractive antibacterial enzymes present shallow, polar, solvent-exposed active sites whose clinical inhibitors are without exception covalent or metal-directed; the β-lactamases are the clearest example. A screen carried out against such a site measures non-specific surface association rather than inhibition, and downstream simulation does not correct this. Pocket-druggability scoring offers a cheap way to test this before committing compute, by asking whether the site has the enclosure and hydrophobicity characteristic of pockets that bind drug-like ligands with high affinity [13,14].

A common remedy for an untrusted docking score is to submit the top-ranked poses to molecular dynamics and retain those that remain bound, on the reasoning that a spurious pose will not survive solvation and thermal motion [15]. A second is to rescore the resulting ensembles with end-point free-energy methods such as MM-PBSA or MM-GBSA [16]. Both substitute one implicit assumption for another: that persistence in the pocket, or a more favourable end-point energy, is evidence of specific engagement. That assumption is rarely tested against a compound known to inhibit the target, because such a compound is not normally simulated; it is not in the hit list, since the scoring function did not place it there.

We therefore did two things differently. First, we selected targets by pocket-druggability triage before screening, retaining only sites that scored as druggable and that also reproduced their own co-crystal ligand pose on redocking. This yielded four *Escherichia coli* enzymes spanning distinct chemistries and mechanisms: FabI, the enoyl-ACP reductase that catalyses the rate-determining step of fatty-acid elongation and the target of triclosan [17]; LpxC, the zinc-dependent deacetylase that commits UDP-GlcNAc to lipid A biosynthesis and remains one of the few Gram-negative-selective targets without a marketed inhibitor [18]; DHFR, the target of trimethoprim, against which resistance is now dominated by acquired *dfr* genes encoding insensitive variants [19]; and GyrB, the ATPase subunit of DNA gyrase and the binding site of the aminocoumarin novobiocin [20].

Second, we carried a reference inhibitor of established activity through the identical pipeline alongside the docking hits at each target, so that every computational verdict could be read against a compound known to work. This converts the study from a ranking exercise into an exploratory comparison against a compound of established activity. The docking hits themselves have no established activity at these targets and are not negative controls, so all comparisons below are descriptive within this small panel. We report where docking, pocket occupancy, contact-loss time and MM-GBSA each failed to separate the reference from the hits, identify the one measure that separated them in this panel, and use that benchmark to nominate, as hypotheses, the hits that warrant experimental testing. Because all four targets are Gram-negative enzymes, we note at the outset that any candidate emerging from such an analysis must additionally cross the outer membrane and evade efflux, constraints that govern Gram-negative antibacterial activity largely independently of target affinity [21].

## 2. Results

### 2.1. Pocket-Druggability Triage of Candidate Targets

Seven candidate structures were assessed with fpocket [13] using the druggability model of Schmidtke and Barril [14]. Four passed and were taken forward (Table 1). All four are *E. coli* enzymes, so the panel varies the target while holding the organism fixed. Each docking protocol reproduced its own co-crystal ligand pose to 0.50–0.69 Å, well inside the conventional 2.0 Å success criterion.

Cofactor handling dominated the triage. Stripping every HETATM record empties the cofactor cavity, which fpocket then scores as an excellent drug pocket: *E. coli* thioredoxin reductase scored 0.993 in that state, on a pocket centred 4.0 Å from the FAD it had just lost, against 0.154 with FAD present. Because this artefact inverts the ranking of a target panel and is signalled by no warning, cofactors were retained throughout (Section 4.1). Thioredoxin reductase was accordingly excluded (0.15 and 0.01 for the *E. coli* and *S. aureus* catalytic cysteine sites); MurA was borderline (0.33) and was not pursued.

### 2.2. Docking Rank of the Reference Inhibitors

Best affinities were comparable across the four targets (−10.4 to −11.3 kcal/mol). The reference inhibitors did not rank with them (Table 2).

Only FabI recovered its reference in the top 5%, and FabI is the one target whose structure is a co-crystal with that compound, so the pocket is shaped around it. Methotrexate, a picomolar DHFR inhibitor, ranked in the 21st percentile; novobiocin was buried at the 28th percentile even though the GyrB structure used here is a co-crystal of its close analogue clorobiocin.

The top of each list was instead occupied by large, promiscuous drugs. Pooling the top ten compounds at each target gives a median of 37 heavy atoms against a library median of 22, and the same molecules recur across mechanistically unrelated targets (lumacaftor ranked first at both DHFR and GyrB). Affinity correlated with heavy-atom count at every target (Spearman *ρ* = −0.34 at FabI, −0.56 at DHFR, −0.63 at GyrB, −0.78 at LpxC; all n > 2060), confirming that the ranking is substantially a size ranking.

### 2.3. Complex Stability, Pocket Occupancy and Contact-Loss Time

**Figure 1:**
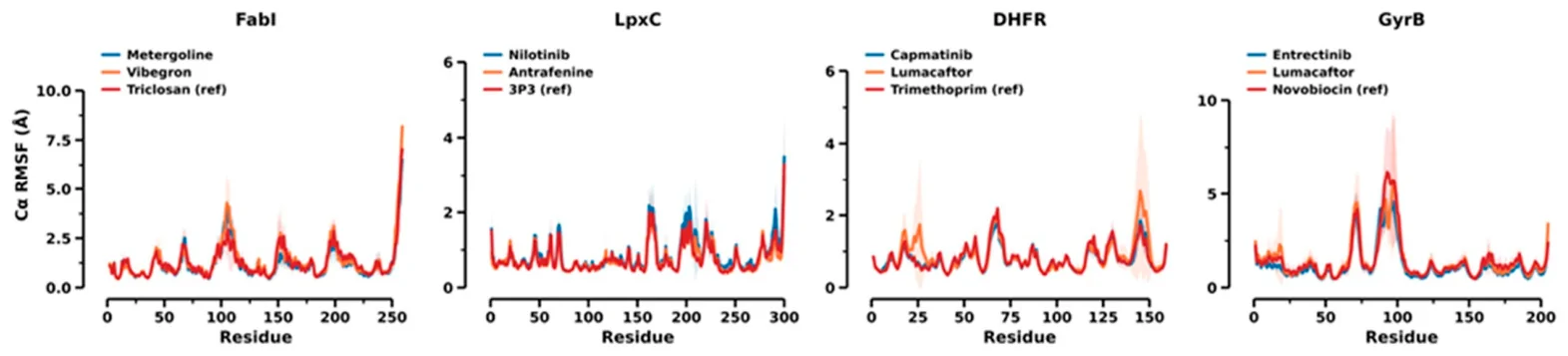
Per-residue Cα RMSF, unsmoothed, grouped by target. Lines are the mean of three replicates, bands ±1 SD. The GyrB trace identifies the rebuilt loop as the dominant contribution to that target’s elevated backbone RMSD; elsewhere the traces for the three ligands at a given target are nearly superimposable, indicating the ligand does not reshape the fold. Residues are numbered sequentially along each simulated model, so the GyrB peak near residue 95 corresponds to the segment deposited as 101–115.

All twelve systems were structurally stable over 100 ns. Protein backbone RMSD was 1.2–2.7 Å for FabI, LpxC and DHFR, and 3.3–4.3 Å for GyrB (Figure S1), and the change in each metric between the first and second halves of the sampling window was small for most complexes (Figure S3, Table S5), although agreement between the two halves is limited evidence of convergence (Section 3.5). The radius of gyration was constant in every complex, including GyrB at 1.64–1.70 nm (Figure S2), confirming that no protein unfolded and that the rebuilt GyrB chain does not separate under periodic boundary conditions. The elevated GyrB backbone RMSD is localised rather than global: per-residue RMSF identifies the rebuilt loop as the dominant contribution, while the remainder of each fold is comparably rigid across targets and largely unaffected by which ligand is bound (Figure 1).

**Figure 2:**
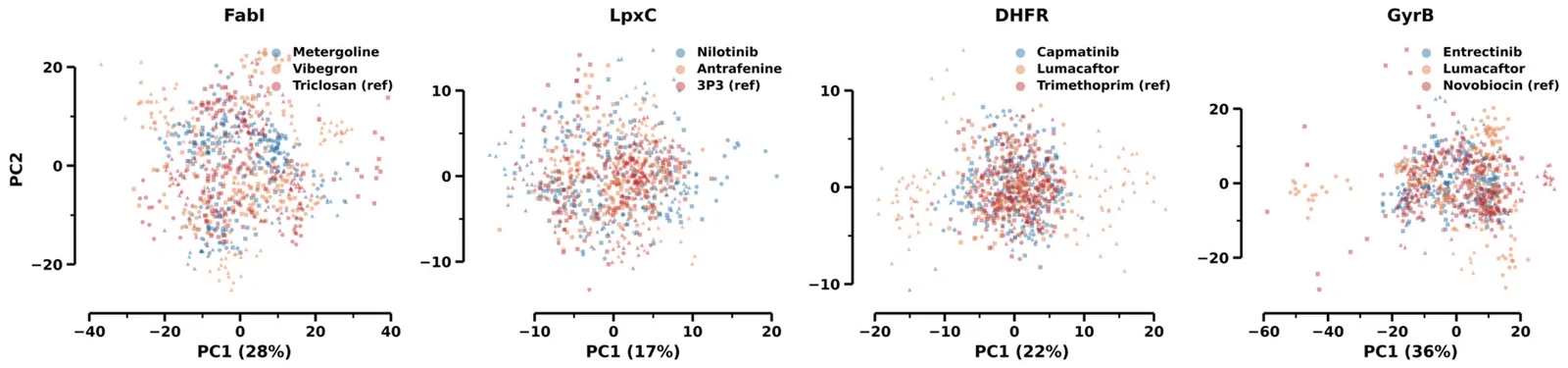
Projection of the Cα coordinates onto the first two principal components over the equilibrated window; the three replicates of each complex are drawn with different markers. Percentages on the abscissa are the variance captured by PC1, averaged over the complexes of that target.

Principal component analysis of the Cα coordinates, computed separately for each complex, describes the conformational space sampled in each simulation (Figure 2). Replicates of the same complex overlap in the plane of the first two components, and the three ligands at a given target cover similar regions of that plane. Because each complex has its own basis, this overlap is a qualitative indication that no replicate diverged into a distinct state; it does not show that the complexes sample an identical ensemble. Together with the near-superimposable RMSF profiles (Figure 1), it argues against gross differences in protein conformation as the origin of the ligand differences reported below, but does not exclude subtler ones.

**Figure 3:**
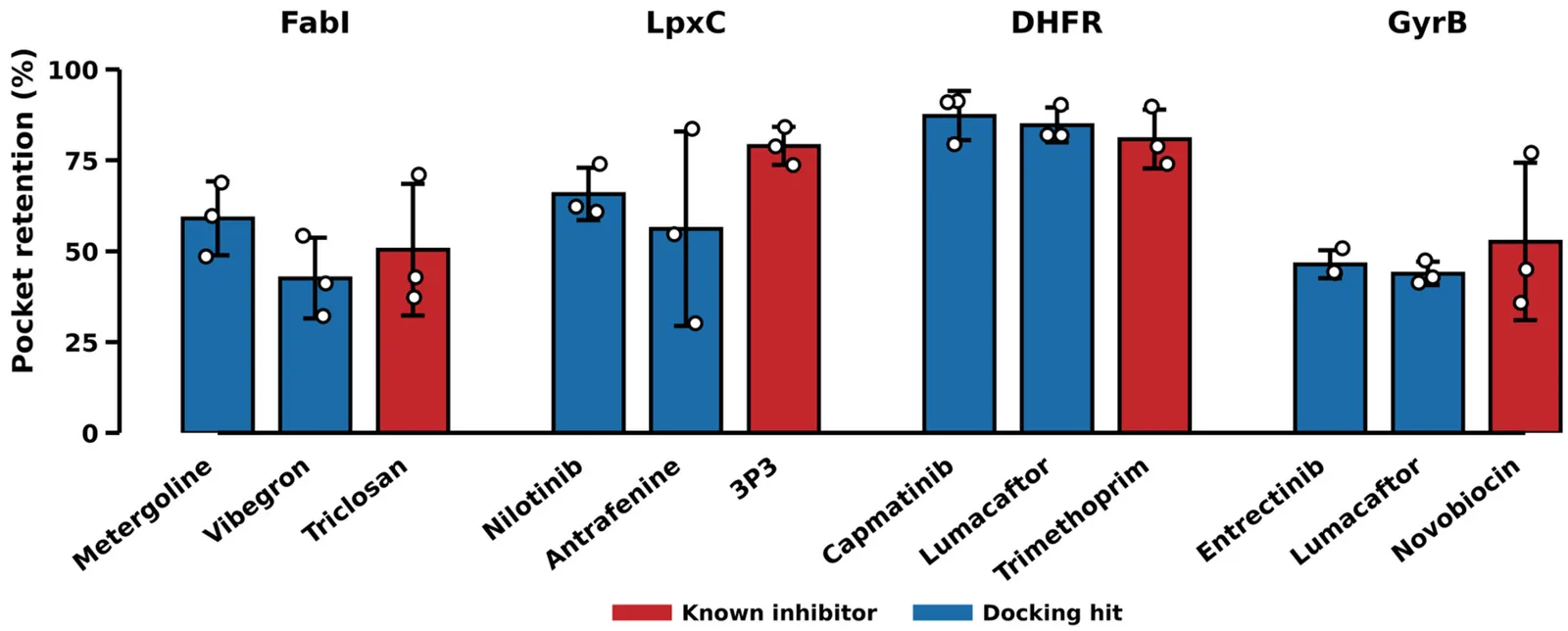
Pocket retention (percentage of initial site contacts maintained), means over 25–100 ns with the three replicate values overlaid as open circles; error bars are ±1 SD between replicates. Occupancy does not separate reference inhibitors (red) from docking hits (blue); at DHFR, the reference ranks last of the three.

Pocket retention over 25–100 ns ranged from 43% to 87% across the twelve complexes (median 58%; Figure 3), and no complex lost all of its initial contacts. Occupancy therefore does not separate reference inhibitors from docking hits: at DHFR, trimethoprim (80.9 ± 8.1%) ranks third of three, behind capmatinib (87.3 ± 6.8%) and lumacaftor (84.8 ± 4.8%), all within one standard deviation.

**Figure 4:**
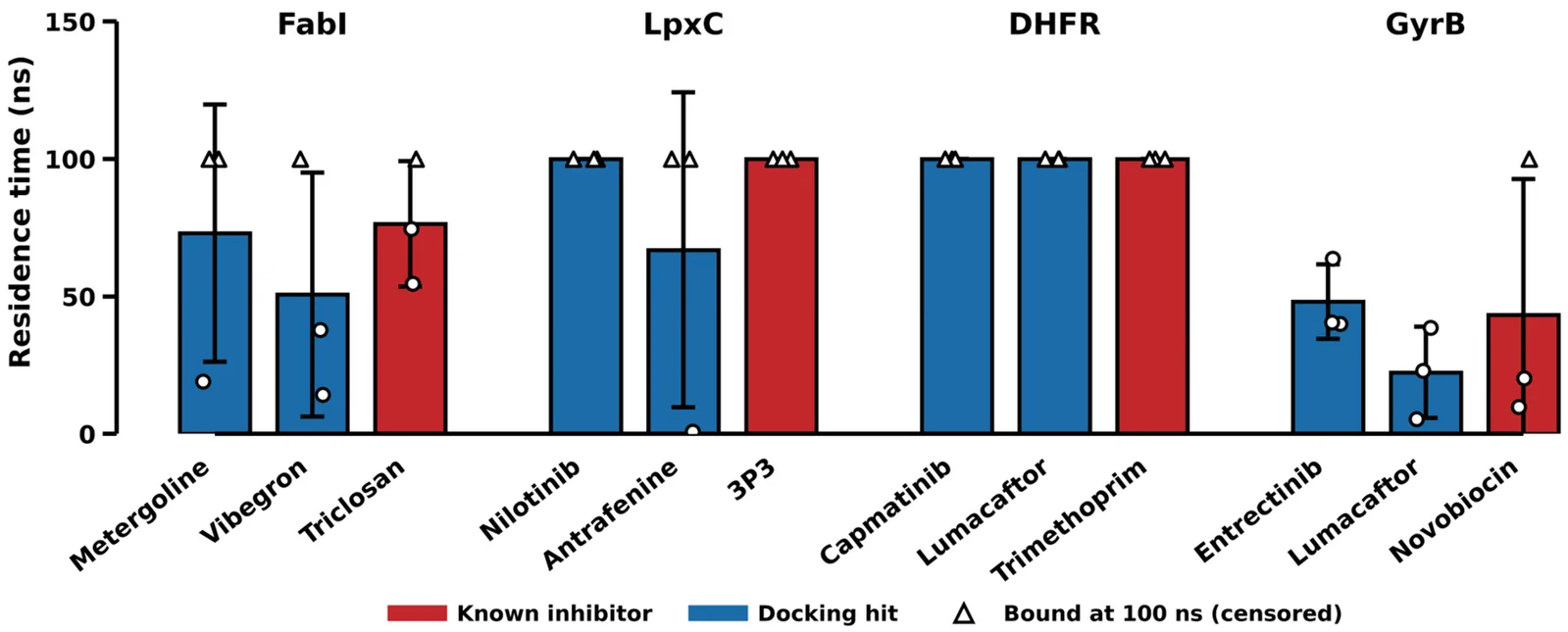
Contact-loss time (plotted as “residence time”), defined as the first sustained (≥5 ns) drop of pocket retention below 50% of its initial value (Section 4.6). Open triangles mark replicates still bound at 100 ns, which are right-censored rather than counted as contact-loss events; open circles mark measured events.

Contact-loss time separates targets rather than compounds (Figure 4). At DHFR, every ligand, reference and hit alike, had not lost its contacts by 100 ns (right-censored), and at LpxC eight of nine replicates were likewise censored. At GyrB, no replicate of either docking hit survived the trajectory (contact loss at 5–64 ns) and novobiocin held in only one of three. One antrafenine replicate at LpxC met the contact-loss criterion at 1 ns. Because loss of initial contacts can reflect rearrangement of the pose within the site as well as true dissociation, the measure is reported as contact-loss time rather than residence time, with events and censored replicates given separately (Table 3). It identifies which sites retain a ligand’s initial contacts at all, but within a target it does not separate the reference inhibitor from the hits.

### 2.4. Persistence of Protein–Ligand Hydrogen Bonds

Persistent protein–ligand hydrogen bonding separates the reference from the docking hits at three of four targets in this panel (Table 3), although at GyrB the separation rests largely on a single replicate (Section 3.5).

**Figure 5:**
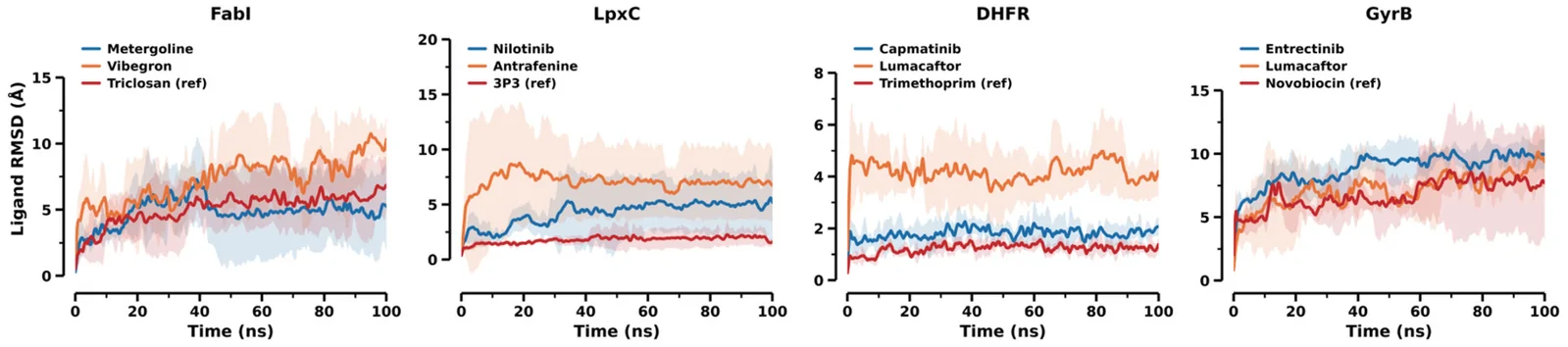
Ligand RMSD after superposition on the binding-site residues, measuring drift of the pose within its own site (Section 4.6). The reference inhibitor is the least mobile ligand at DHFR, LpxC and GyrB.

**Figure 6:**
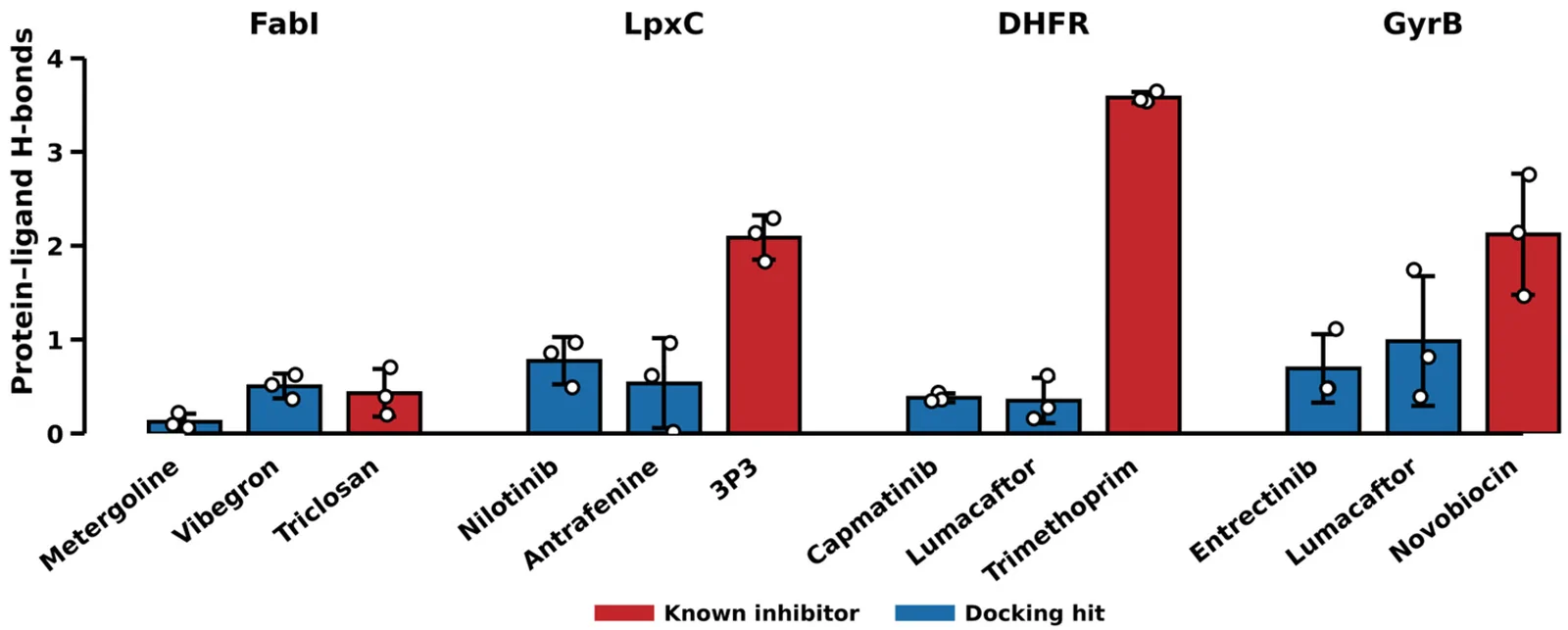
Persistent protein–ligand hydrogen bonds (donor–acceptor 3.5 Å, angle 120°), mean over 25–100 ns with replicate values overlaid. This is the one metric that separates the reference inhibitor from both docking hits at DHFR, LpxC and GyrB. FabI is the exception (Section 2.7), where triclosan binds by π-stacking on NAD^+^.

At DHFR, LpxC and GyrB, the reference inhibitor was the least mobile ligand by site-superposed RMSD (Figure 5) and formed 2–9-fold more persistent hydrogen bonds than either docking hit (Figure 6). Trimethoprim’s 3.6 hydrogen bonds are consistent with its crystallographic binding mode, in which the protonated diaminopyrimidine hydrogen bonds to Asp27 and to conserved waters. A contact-area scoring function does not reward this charge-driven interaction, which plausibly contributes to its rank of 762.

The separation is orthogonal to occupancy. At DHFR, the two hits are retained better than trimethoprim while forming one-ninth as many hydrogen bonds: they occupy the site without engaging it.

Resolving the count by residue qualifies this result rather than reinforcing it (Table S7, Figure S6). Trimethoprim holds three residues (Ile5, Ile94 and Asp27) in 95–99% of frames in every replicate. The LpxC reference has one residue above 50% occupancy (0.65), and novobiocin does not: its 2.1 hydrogen bonds are distributed over 13 residues on average with no single partner exceeding 31% occupancy, so at GyrB the reference forms about as many hydrogen bonds as trimethoprim does but redistributes them rather than holding a defined set. Per-residue occupancies sum to the totals of Figure 6 in every case, so the two analyses are consistent; what differs is whether the bonds are made to the same residues throughout. On this stricter criterion, only DHFR, and partially LpxC, show the residue-specific engagement that the aggregate count implies, which is independent support for treating GyrB as the weakest of the four targets (Section 3.5). Residue-level partner statistics are summarised for every complex in Table S7, but individual partner identities are named there only for trimethoprim; they were not tabulated for the other compounds (Section 3.5).

**Figure 7:**
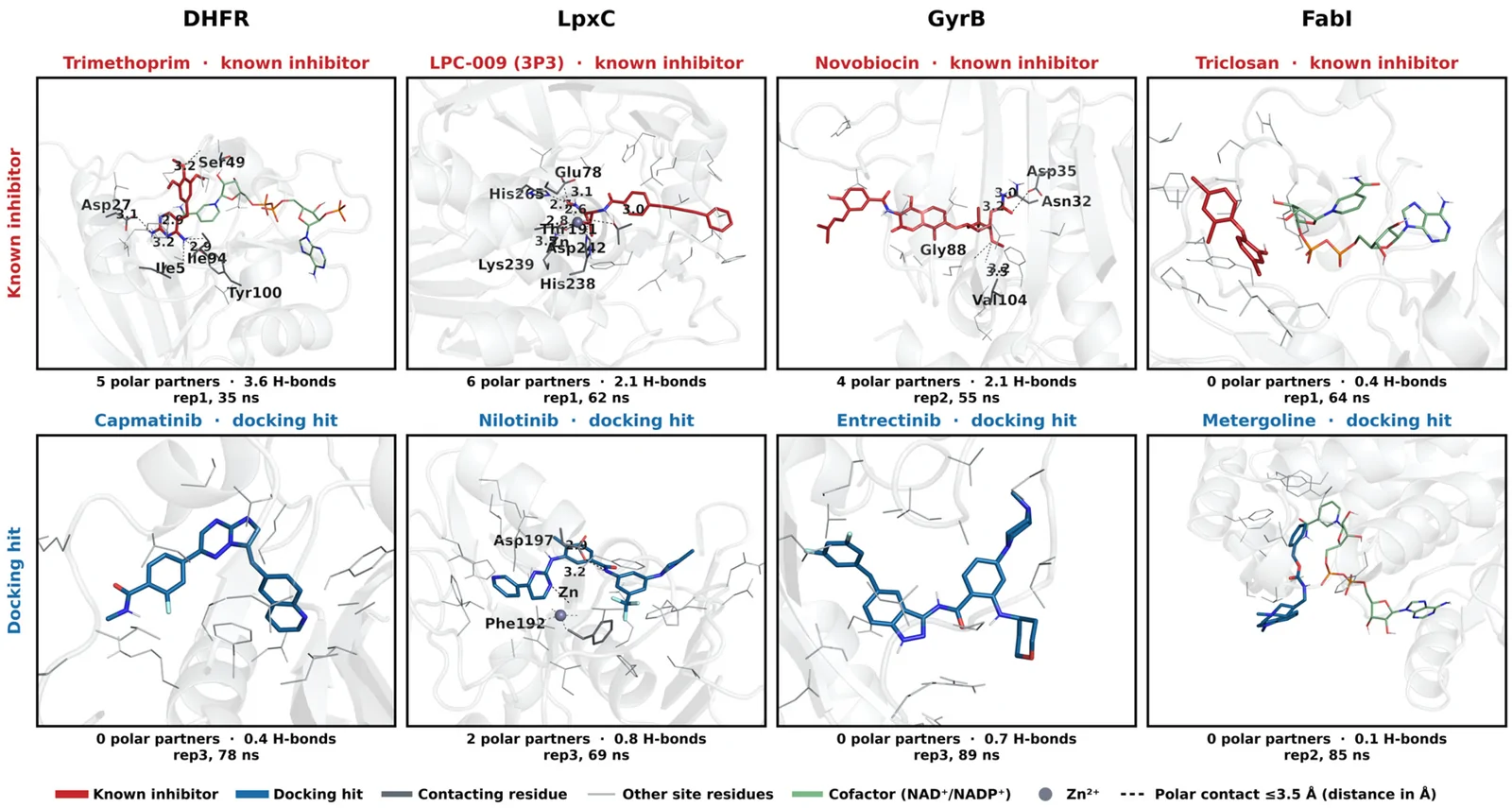
Binding modes in representative MD frames. Columns are targets; the known inhibitor (red) is shown above the docking hit (blue). Frames are the median replicate and median ligand-RMSD frame of the equilibrated window, so no compound is shown in its most favourable frame (Section 4.9). Residues making a polar contact (≤3.5 Å) with the ligand are drawn as labelled sticks with the contact dashed; all other residues lining the site are drawn as thin lines to show the enclosure. Cofactors (NADP^+^, NAD^+^) are pale green. Panel footers give the single-frame polar-partner count and, for comparison, the trajectory-averaged hydrogen-bond occupancy from Table 3. In the FabI panel, triclosan has left its crystallographic π-stacking pose on the NAD^+^ nicotinamide; this is characteristic of the ensemble rather than an unrepresentative frame, matching its 50.4 ± 18.2% retention and 5.59 Å mean ligand RMSD (Section 2.7).

The same contrast is visible structurally (Figure 7). Counting the residues that make a polar contact with the ligand in a representative frame reproduces the trend independently of the time-averaged hydrogen-bond analysis: five partners for trimethoprim against none for capmatinib, six for LPC-009 against two for nilotinib, and four for novobiocin against none for entrectinib. The docking hits sit in the same cavities, enclosed by the same residues, without engaging them.

### 2.5. MM-GBSA Rescoring

**Figure 8:**
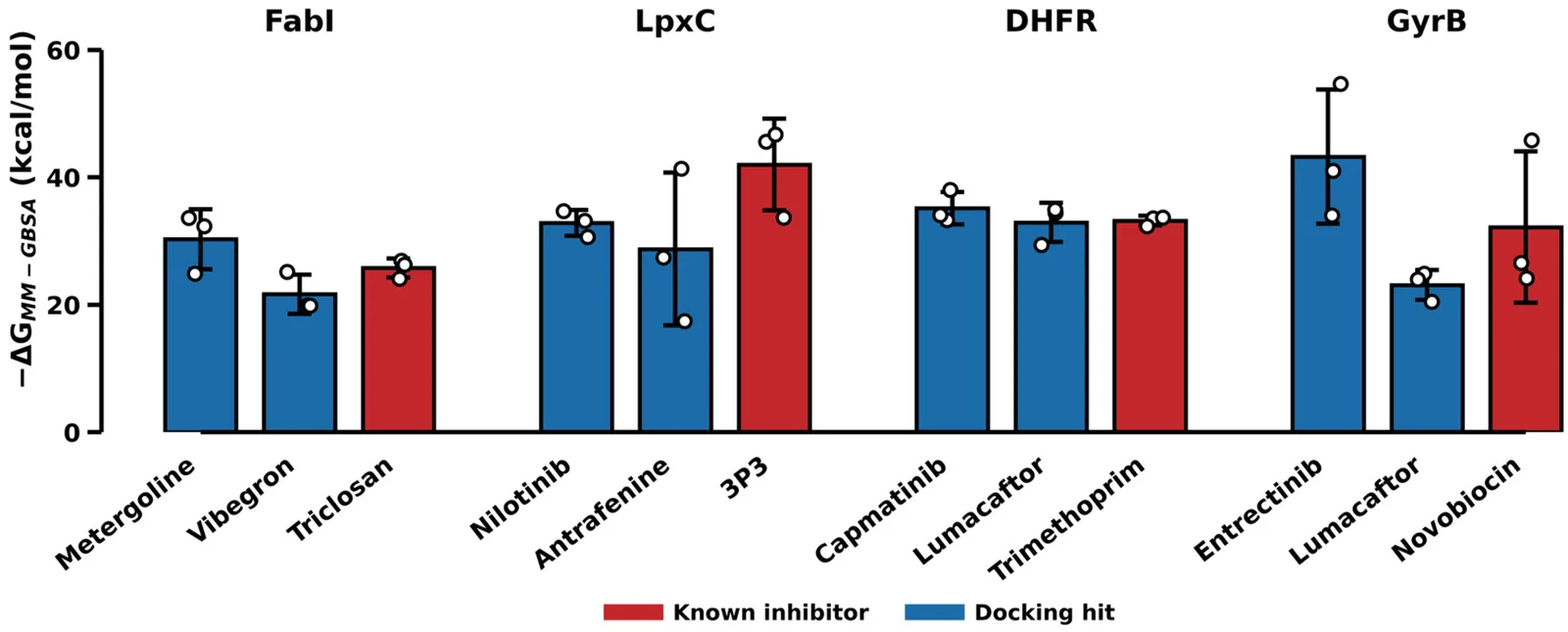
MM-GBSA effective interaction energy per complex, plotted as −ΔG so that a taller bar is a more favourable predicted binder. Bars are means over three replicates with the replicate values overlaid; error bars are ±1 SD. The known inhibitor (red) is the most favourable compound only at LpxC.

Because end-point free-energy rescoring is the usual next step after a docking screen, we computed single-trajectory MM-GBSA effective interaction energies (no entropy term) for every replicate [26,27] (Figure 8, Table 4). They do not recover the reference inhibitors, and with three replicates most differences between compounds at a target lie within replicate uncertainty.

Only at LpxC is the reference inhibitor the most favourable compound by point estimate. At DHFR, capmatinib (−35.2) scores better than trimethoprim (−33.2) despite forming one-ninth as many hydrogen bonds; at GyrB, entrectinib (−43.2) scores 11 kcal/mol better than novobiocin; at FabI, metergoline outscores triclosan. With three replicates, however, the 95% Welch confidence interval of every hit-minus-reference difference includes zero (capmatinib minus trimethoprim −2.0 kcal/mol, 95% CI −7.7 to +3.7; metergoline minus triclosan −4.5, −15.0 to +6.0; nilotinib minus the LpxC reference +9.2, −7.2 to +25.6; entrectinib minus novobiocin −11.0, −36.6 to +14.6; Table S3), so these data establish neither a ranking nor equivalence of affinity.

Decomposing the energy identifies the cause (Table S3). At three of four targets, the compound with the largest van der Waals term is the one with the most favourable total, so MM-GBSA reproduces the same buried-contact-area preference that drives the docking score rather than correcting it. The exception is LpxC, where the reference does not win on contact area; nilotinib has the larger van der Waals term (−49.7 versus −38.1 kcal/mol). The reference’s gas-phase electrostatic term is the largest in the study (−71.5 kcal/mol, against −16.5 for nilotinib and −21.2 for the next largest compound), consistent with hydroxamate chelation of the catalytic Zn^2+^, but this is largely cancelled by polar solvation (ΔGGB +74.2 kcal/mol). The net polar contribution, ΔEelec + ΔGGB, is +2.7 kcal/mol for the reference against +23.5 for nilotinib, and it is this combined term that offsets nilotinib’s 11.6 kcal/mol van der Waals advantage (Table S3). The LpxC advantage of the reference is therefore a property of the net polar balance and not of the electrostatic term alone; because Zn^2+^ coordination was not treated with a multi-centre bonded metal model (Section 3.5), it should be regarded as qualitative. In this panel, end-point rescoring recovers the reference only where a charge-driven, metal-directed interaction is present.

Replicate spread is also large where the ligand is mobile (±12.0 kcal/mol for antrafenine and ±11.9 for novobiocin), so the ordering at those targets is not stable even across replicates of the same complex.

In this panel, MM-GBSA therefore behaved as a more expensive version of the docking score rather than as an independent check on it. This is consistent with the broader literature on end-point methods, and it is the reason we do not present it as a rescoring solution.

### 2.6. Water-Mediated Bridges

The hydrogen-bond count of Section 2.4 is a direct protein–ligand criterion and is therefore blind to contacts relayed through a bridging water. This matters here because trimethoprim’s crystallographic mode is partly water-mediated. We counted, in every frame, the waters simultaneously within 3.5 Å of a ligand polar atom and of a protein polar atom (Section 4.7).

**Figure 9:**
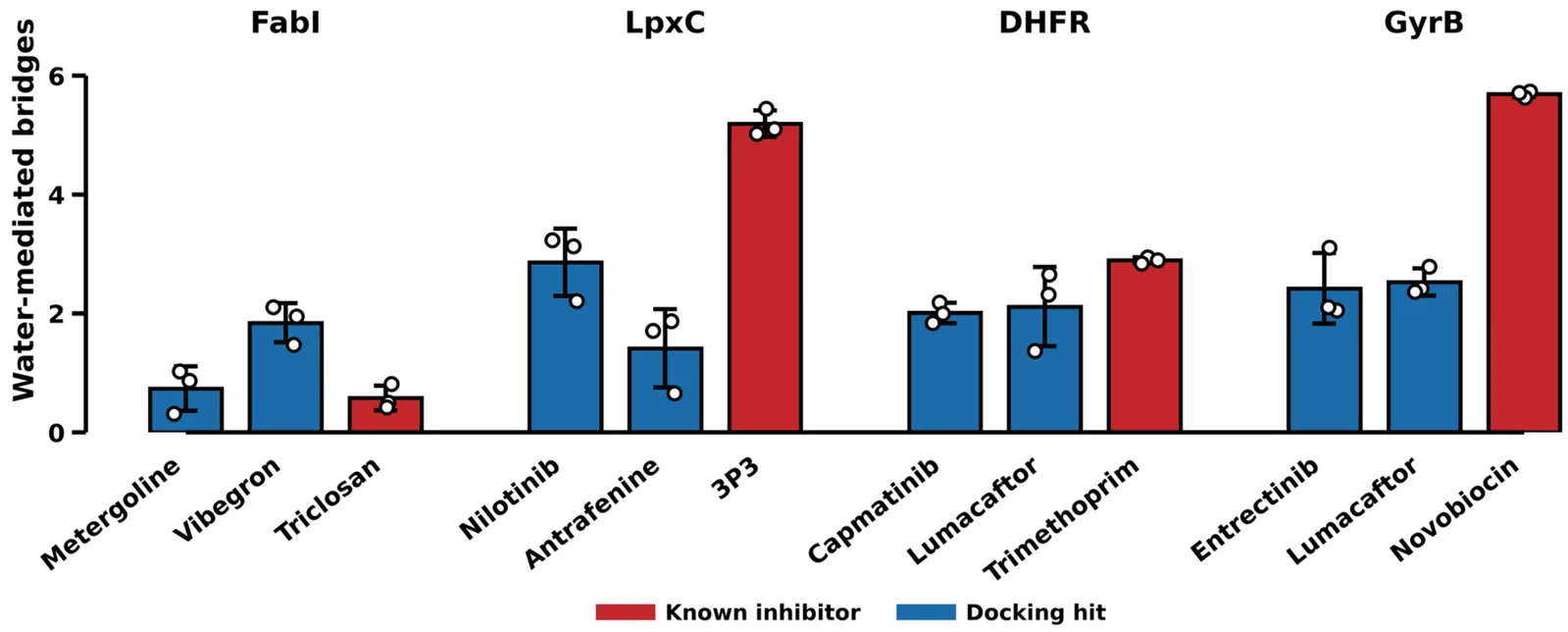
Water-mediated bridges per frame: waters simultaneously within 3.5 Å of a ligand polar atom and a protein polar atom, averaged over 25–100 ns. Bars are means over three replicates with replicate values overlaid; error bars ±1 SD. The known inhibitor forms the most bridges at DHFR, LpxC and GyrB, and the fewest at FabI.

Water bridges separate the two classes at the same three targets, with the same exception (Figure 9). The reference forms most bridges at DHFR (2.9 ± 0.1 versus 2.0 and 2.1), LpxC (5.2 ± 0.2 versus 2.9 and 1.4) and GyrB (5.7 ± 0.1 versus 2.4 and 2.5), and fewest at FabI (0.6 versus 0.7 and 1.8). An independent measurement computed from the solvent rather than from the protein reproduces both the discrimination and its single failure, which strengthens the evidence beyond either count alone. A third descriptor, the specificity of the contact set (the fraction of contacted residues held in at least 80% of frames), favours the reference at DHFR and LpxC only, discriminating at two of four targets; it is reported in Table S6 and Figures S4 and S5 rather than in the main text because it is the weaker of the three.

GyrB is the target where the two measures differ most, although it remains exploratory (Section 3.5). Novobiocin’s hydrogen-bond advantage rests substantially on one replicate (Section 3.5), but its water-bridge count is 5.7 ± 0.1 against 2.4 ± 0.6 and 2.5 ± 0.2 for the two hits, a separation of more than twofold with a replicate spread an order of magnitude smaller than the gap. At this target, the water-bridge count is more reproducible than the hydrogen-bond count and supports the same ordering, but this does not remove the fragility of GyrB as a target.

### 2.7. The FabI Exception

FabI does not follow the pattern. Triclosan formed few hydrogen bonds (0.4), was not the least mobile ligand, and had the widest replicate spread in retention (43, 71, 37%). This is consistent with its binding mode: triclosan binds FabI principally by π-stacking on the nicotinamide ring of NAD^+^, with a single hydroxyl contact to Tyr156 [23], so a protein–ligand hydrogen-bond count is structurally blind to the interaction that matters.

FabI also showed among the lowest retention of the four targets despite the second-highest druggability score, indicating that pocket-shape scores and interaction persistence measure different things. Water bridges fail at FabI for the same structural reason as direct hydrogen bonds (Section 2.6): a π-stacking interaction is invisible to any polar criterion, mediated or not.

### 2.8. Candidate Compounds for Experimental Testing

Benchmarking each hit against a known inhibitor carried through the same pipeline allows the twelve complexes to be ranked by how closely a hit reproduces reference-level behaviour (Table 5). Selection used two criteria, specified after the simulations and applied identically at every target: a hit was considered if it kept its initial contacts to 100 ns (contact-loss time right-censored) in at least two of three replicates, and the better of the two hits at that target was nominated if it was also ahead of its sibling on pocket retention, ligand RMSD and MM-GBSA. Both criteria describe pose persistence and approximate energy, which Section 2.3 and Section 2.5 show do not, by themselves, establish specific binding, so the nominations are hypotheses for experimental testing and not predictions of inhibition. Hydrogen-bond persistence was not used for selection, and the three nominated compounds form far fewer hydrogen bonds than their references (0.1–0.8 versus 0.4–3.6; Table 3). Three compounds are therefore proposed as candidates for experimental testing.

Capmatinib at DHFR ranks highest of the three on these criteria. It gave the highest pocket retention of all twelve complexes (87.3 ± 6.8%), the second-lowest ligand RMSD after trimethoprim itself (1.86 ± 0.34 Å), and kept its initial contacts through the full 100 ns in all three replicates. Its MM-GBSA energy cannot be distinguished from trimethoprim’s at this sample size (−35.2 ± 2.5 versus −33.2 ± 0.7 kcal/mol; difference −2.0 kcal/mol, 95% CI −7.7 to +3.7, Welch, n = 3); this is absence of evidence for a difference, not evidence of equivalent affinity. Capmatinib is an approved MET kinase inhibitor, and its aminoquinoline core occupies the folate pocket without reproducing trimethoprim’s diaminopyrimidine hydrogen-bond network (0.4 versus 3.6 bonds), which suggests, as a hypothesis, binding driven by shape complementarity rather than by reproduction of the reference mechanism.

Metergoline at FabI has better mean values than triclosan for retention (59.1 versus 50.4%), ligand RMSD (5.15 versus 5.59 Å) and MM-GBSA (−30.3 ± 4.7 versus −25.8 ± 1.5 kcal/mol; difference −4.5 kcal/mol, 95% CI −15.0 to +6.0), but it forms fewer hydrogen bonds (0.1 versus 0.4) and the differences are within replicate spread. Because triclosan is itself poorly retained at this target (Section 2.7), metergoline should be read as showing no detectable difference from a weakly behaved reference rather than as strongly validated.

Nilotinib at LpxC kept its initial contacts through the full 100 ns in all three replicates and was the better-behaved of the two LpxC hits, but its MM-GBSA energy is 9.2 kcal/mol less favourable than the reference by point estimate (95% CI −7.2 to +25.6) and its pose drifts further (4.71 Å), so it is proposed as the weakest of the three.

GyrB yielded no candidate. Both hits lost their initial contacts early (mean contact-loss times 48 and 22 ns, no censored replicates) and drifted 7.6–9.3 Å from their docked poses, and neither approached novobiocin on any measure.

On ligand efficiency, no candidate exceeds its reference (Table 5), so the favourable absolute MM-GBSA values reflect the size bias documented in Section 2.2 rather than superior complementarity. These are computational nominations for experimental testing, not validated inhibitors.

## 3. Discussion

The practical result is that the two axes most often used to triage a virtual screen, the docking score and whether the pose survives simulation, both failed to identify the compounds known to work, while a third, the persistence of specific hydrogen bonds, separated the reference from the hits at three of four targets in this small panel.

### 3.1. Systematic Bias in the Docking Score

Affinity correlated with heavy-atom count at every target (Spearman *ρ* up to −0.78), and the same handful of large drugs surfaced at the top of mechanistically unrelated lists. Compounds such as trimethoprim and novobiocin bind through small, highly specific, charge- and water-mediated networks that contribute little buried non-polar surface, and they are ranked accordingly, in the middle of the library. This is a known property of empirical scoring functions, but its consequence for repurposing is usually invisible, because the known actives are not in the hit list and so are never scored.

### 3.2. Pocket Occupancy as a Triage Criterion

Pocket retention is a common proxy for “the pose is real”. At DHFR every complex, and at LpxC all but one replicate, kept its site contacts for the full 100 ns, including compounds with no established relationship to the target: capmatinib and lumacaftor were retained at DHFR as well as trimethoprim, and better by mean occupancy. On a genuinely druggable pocket, a large hydrophobic molecule will sit in a hydrophobic cavity for 100 ns whether or not it inhibits anything. Where ligands did leave, they left indiscriminately: at GyrB, hits and reference dissociated alike. In neither regime does occupancy separate the classes. Occupancy tests whether the site can hold a ligand, not whether the ligand engages the residues responsible for inhibition.

### 3.3. End-Point Rescoring and Buried-Area Bias

MM-GBSA is commonly applied when a docking score is not trusted. It failed here for the same reason the docking score did. Its ranking follows the van der Waals term at three of four targets (Table S3), so it reproduces the buried-area preference rather than correcting it: the reference inhibitor is the most favourable compound at one target of four. The single success is also informative: at LpxC the reference wins on a Zn^2+^-chelating electrostatic term rather than on shape complementarity, which suggests, tentatively, that end-point rescoring may be more informative for charged, metal-directed chemistry than for the remaining compounds in this panel, although that electrostatic term is largely offset by polar solvation (Section 2.5). Where the ligand is mobile, the replicate spread reaches ±12 kcal/mol, larger than the differences being ranked. A more costly calculation based on the same physics therefore does not supply the missing specificity.

### 3.4. Specific Versus Gross Interaction Measures

What discriminated was the count of persistent hydrogen bonds, a measure of specific rather than gross interaction. We would not present this as a validated scoring method on the strength of four targets and one active each; that requires decoys and enrichment statistics. With one active per target, hydrogen-bond persistence has not been shown to be a general predictive metric, and the result is better read as an observation specific to these trajectories. But the direction is consistent, the margins are large (2–9-fold), and the mechanism is interpretable. At FabI, where triclosan binds mainly by π-stacking on NAD^+^, hydrogen-bond metrics are not expected to be informative, and we do not interpret hydrogen-bond counts at that target as evidence for or against binding; a different descriptor, such as stacking geometry, would be required. The general claim is not “count hydrogen bonds” but “measure the specific interaction the chemistry implies, not the gross one”.

For practice, we draw two conclusions. Druggability triage before screening is cheap, takes minutes per structure, and changes which targets are worth the computational cost, provided cofactors are retained, since stripping them inverts the ranking (Section 2.1). And a reference inhibitor should be carried through the entire pipeline alongside the hits: it costs one extra simulation per target, and it was the only element of this study that revealed the screen was not recovering actives. Without it, the twelve trajectories reported here would have supported a confident claim that two promiscuous kinase-directed drugs are established DHFR inhibitors, on the strength of 87% and 85% pocket occupancy alone. With it, the same data place capmatinib at an appropriate level of confidence: a stable binder worth testing (Section 2.8) that does not reproduce the reference’s interaction pattern and cannot be assumed to share its mechanism.

### 3.5. Limitations

GyrB gives the weakest contrast of the four targets. Its missing loops were rebuilt (Section 4.2) and the resulting fold is stable, but the modelled 15-residue segment remains the most flexible part of the protein and the backbone RMSD (3.3–4.3 Å) is correspondingly higher than at the other three targets. More importantly, novobiocin itself is poorly retained: it holds its pose for the full 100 ns in one replicate but leaves after 20 and 10 ns in the other two, giving the largest replicate spread in the study (52.7 ± 21.7%). Its hydrogen-bond advantage over the two hits (2.1 versus 1.0 and 0.7) therefore rests substantially on a single replicate and should be read as suggestive rather than established on that metric alone; the water-bridge count at this target is far more reproducible (5.7 ± 0.1 versus 2.4 ± 0.6 and 2.5 ± 0.2, Section 2.6) and supports the same ordering. We report GyrB because excluding a target after seeing its result would bias the comparison, not because it supports the conclusion. GyrB conclusions should therefore be regarded as exploratory: with a reference that held its pose in only one of three replicates and a rebuilt 15-residue loop, this target cannot be used to evaluate any descriptor, and the three-of-four result is better read as two firm targets (DHFR and LpxC) and one tentative one.

A further caveat is specific to GyrB: the loop defect was found after the library screen, so the GyrB ranks in Table 2 come from the unrebuilt structure (Section 4.2), even though every GyrB simulation uses the rebuilt one. Novobiocin’s 28th percentile therefore applies to the deposited coordinates as distributed, which is how such a screen would ordinarily be run, rather than to the rebuilt receptor. We did not re-screen the library against the rebuilt structure, so we cannot say whether its rank would shift.

The design is a contrast, not a benchmark. One reference inhibitor per target against two hits shows a difference within this panel, but an enrichment study with property-matched decoys and multiple actives per target would be required to claim that MD-derived hydrogen bonding is a general rescoring method. In particular, with a single active at FabI, we cannot distinguish whether the failure there is specific to triclosan’s stacking mode or general to the target. The docking hits have no established activity at these targets and so cannot serve as negative controls; differences between hits and references are descriptive comparisons within a panel of twelve complexes and not a measurement of discrimination between actives and inactives.

Other limitations are methodological. NADP^+^ used Gasteiger rather than AM1-BCC charges because AM1-BCC failed to converge for this 73-atom trianion; mixing charge schemes within one binding site may introduce an electrostatic imbalance, and the sensitivity of the results to this choice was not tested, so electrostatic quantities at DHFR (hydrogen-bond occupancy and the MM-GBSA electrostatic terms) should be interpreted with caution. Systems were neutralised with counter-ions only, without physiological salt, whereas MM-GBSA used 0.15 M implicit salt; the dynamics were therefore run at near-zero ionic strength, which may over-stabilise exposed salt bridges and polar contacts, particularly in charged and metal-binding systems such as LpxC. Three replicates of 100 ns support a comparison of persistent interactions but not rigorous free-energy estimates. MM-GBSA was computed (Section 2.5) as an approximate energy estimate, without an entropy term and with the known limitations of end-point methods; alchemical free-energy calculations were not attempted. Hydrogen bonds were counted with a fixed geometric criterion (donor–acceptor distance 3.5 Å, angle 120°). Water-mediated bridges, which the direct count cannot see, were quantified separately (Section 2.6) and reproduce the same discrimination; the water-bridge count uses distance criteria without an angular term, so it is an upper bound on strictly bridging hydrogen bonds. No experimental validation was performed. The three candidates of Section 2.8 are proposed on simulation evidence alone and require enzyme-inhibition assays to confirm; for capmatinib, the decisive experiment is selectivity over human DHFR, which its shape-driven binding mode leaves entirely open.

The simulation model itself may contribute to the drift of the reference ligands, notably triclosan and novobiocin, independently of any screening descriptor. The starting poses of the reference complexes, the cofactor and metal parameters, and the key interactions were not independently validated. Zn^2+^ coordination at LpxC was not treated with a multi-centre bonded or polarisable metal model, which is generally needed to hold coordination geometry and polarisation over long trajectories; triclosan–NAD^+^ stacking was not benchmarked against crystallographic distances; and the Gasteiger charges used for NADP^+^ were not tested for sensitivity. Reference-ligand drift should therefore be read as a possible limitation of the model and not necessarily as a failure of the descriptors, and the LpxC electrostatic terms as qualitative.

Sampling and convergence were assessed only through agreement between the first and second halves of the window (Table S5) and the per-replicate values in Table S4; block averages and cumulative averages were not computed, and the principal component analysis was computed per complex and is descriptive. With three replicates, the reported means should not be read as converged ensemble averages, and the MM-GBSA differences carry 95% confidence intervals that include zero at every target. The contact-loss time records loss of initial contacts, which cannot distinguish dissociation from rearrangement of the pose within the site.

Experimental follow-up should include enzyme-inhibition or direct-binding assays on purified DHFR, FabI and LpxC and whole-cell MIC determinations, none of which were part of this computational study. In addition, all four targets are intracellular, and large lipophilic kinase inhibitors such as capmatinib (molecular weight about 412) and nilotinib (about 530) must cross the Gram-negative outer membrane and evade efflux pumps such as AcrAB–TolC. Permeability and accumulation were not part of the triage and may preclude whole-cell activity regardless of target affinity [21].

## 4. Materials and Methods

### 4.1. Target Selection and Druggability Triage

Candidate structures were assessed with fpocket [13] using the druggability model of Schmidtke and Barril [14]. The pocket enclosing each site of interest was identified as the one nearest a co-crystallised ligand or, where none exists, the catalytic residues. Cofactors were retained during the assessment for the reason given in Section 2.1.

### 4.2. Receptor Preparation

The GyrB structure 1KZN lacks residues 101–115 and the 82A–82D insertion. Left unmodelled, its chain enters a simulation as two fragments joined by a 6.4 Å pseudo-bond. A residue-numbering gap check does not detect this, because the numbering is continuous across the break.

This was identified during MD preparation, after the library screen had been run. Both segments were then rebuilt with PDBFixer [28]; the product was verified to contain a single 205-residue chain with no peptide bond longer than 1.5 Å, and redocking clorobiocin into it reproduced the crystallographic pose to 0.69 Å (0.50 Å for the unrebuilt structure, against the same crystallographic reference). The three GyrB complexes advanced to MD were re-docked into the rebuilt receptor, and all GyrB simulations reported here use it. The other three targets have no unmodelled residues in their binding-site regions.

Receptors retained their catalytic cofactors (NAD^+^ for FabI, NADP^+^ for DHFR, Zn^2+^ for LpxC) because these form part of the binding site (triclosan stacks directly on the FabI nicotinamide) and because an empty cofactor cavity otherwise accepts ligands as false positives. Structures were protonated at pH 7.4 and converted to PDBQT.

### 4.3. Library and Docking

The approved-drug subset of DrugBank [29] was filtered to 2070 compounds (Table S1). Search boxes were centred on the co-crystal ligand with a 5 Å pad. Docking used AutoDock Vina (version 1.2.5) [7,8] at exhaustiveness 8 (8280 dockings in total). Each protocol was validated by redocking the native co-crystal ligand and requiring heavy-atom RMSD < 2.0 Å to the crystallographic pose (achieved: 0.50–0.69 Å, Table 1; docking parameters in Table S2).

### 4.4. Reference Inhibitors

Compounds of established activity at each target were carried through the identical MD pipeline: triclosan (FabI), trimethoprim (DHFR), novobiocin (GyrB), and the 3P3G co-crystal inhibitor LPC-009 (LpxC, which has no approved inhibitor; CHIR-090 and ACHN-975 are the literature reference compounds). Additional DHFR actives (methotrexate, pyrimethamine) were docked for the ranking analysis of Section 2.2 but not simulated.

### 4.5. Molecular Dynamics

Twelve complexes (two top-scoring hits plus one reference per target) were simulated in GROMACS 2025.4 [30] with the amber99sb-ildn force field [31] and TIP3P water [32] in dodecahedral boxes with a 1.0 nm solute–boundary margin (23,871–40,766 atoms). Ligands and cofactors were parameterised with GAFF2 [33] and AM1-BCC charges [34] via acpype [35]; NADP^+^, a −3 anion of 73 atoms on which AM1-BCC failed to converge, used Gasteiger charges. Systems were neutralised with Na^+^ counter-ions; no additional salt was added, so the dynamics were run at near-zero ionic strength, whereas MM-GBSA used 0.15 M implicit salt (Section 4.8).

Ligands were rebuilt from the PDBQT SMILES with all hydrogens explicit, because a PDBQT → PDB conversion silently discards non-polar hydrogens and the resulting topology is accepted downstream without error.

Energy minimisation used steepest descent to F_max_ < 1000 kJ mol^−1^ nm^−1^, followed by 100 ps NVT at 310 K (V-rescale thermostat [36], τ_T_ = 0.1 ps, position restraints on solute heavy atoms) and 100 ps NPT at 1 bar (C-rescale barostat [37], τ_P_ = 2.0 ps). Production runs were 100 ns per replicate with a 2 fs timestep, Parrinello–Rahman pressure coupling [38], LINCS constraints on bonds to hydrogen [39], a Verlet cut-off scheme with 1.0 nm real-space cut-offs, and PME electrostatics [40]. Coordinates were written every 200 ps (501 frames per trajectory). Three replicates per complex differed only in initial velocity seed (36 trajectories, 3.6 μs total).

### 4.6. Analysis

Trajectories were made whole and centred (gmx trjconv -pbc mol -centre). Protein RMSD, per-residue RMSF and radius of gyration were computed with the corresponding GROMACS tools against a solute-only run input.

Ligand RMSD was measured after superposition on the binding-site residues rather than on the whole protein. Site residues are in contact with the ligand by construction and therefore always lie in the same periodic image, so this measures drift of the pose within its own site and is immune to the imaging artefacts that whole-protein superposition introduces when a ligand approaches a box boundary.

Pocket retention is the percentage of residues initially within 4.5 Å of the ligand that remain so. Hydrogen bonds were counted with MDAnalysis [41,42] (donor–acceptor 3.5 Å, angle 120°); GAFF atom types carry no element information, so elements were assigned explicitly before analysis; otherwise, ligand donors and acceptors are omitted and every ligand appears to form none.

Contact-loss time is the first time at which pocket retention falls below 50% of its initial value and remains below it for at least 5 ns; the persistence requirement prevents single-frame thermal fluctuations from terminating a trajectory. It records loss of initial contacts, which can reflect rearrangement of the pose within the site as well as dissociation, and is not a dissociation or residence time. Replicates that never cross are right-censored at 100 ns and reported as such rather than as measured events; event times and censoring are reported separately, and no mean is computed over censored values.

All other reported values are means over the post-equilibration window (25–100 ns) across three replicates, quoted as mean ± SD between replicates, where SD is the sample standard deviation (n = 3, denominator n − 1). Error bars and shaded bands in all figures use the same convention. In the time-series figures (Figure 5 and Figures S1 and S2), the plotted mean trace carries a 1 ns running mean applied for legibility only; the ± SD band is computed from the unsmoothed replicates, and every value quoted in the text and tables is from the unsmoothed data. No smoothing is applied to the per-residue RMSF (Figure 1), where the abscissa is residue index rather than time. Convergence of every reported metric over the first and second halves of the sampling window is shown in Figure S3; two-half agreement is a limited test of convergence (Section 3.5).

### 4.7. Contact Occupancy, Water Bridges and Essential Dynamics

Per-residue contact occupancy is the fraction of equilibrated frames (sampled every 400 ps) in which any heavy atom of the residue lies within 4.0 Å of a ligand heavy atom. A residue is termed a *core* contact when its occupancy is ≥0.80. Contact specificity is the ratio of core contacts to all residues contacted in more than 5% of frames, and distinguishes a ligand that holds a few residues persistently from one that grazes many transiently.

A water bridge is a water oxygen simultaneously within 3.5 Å of a ligand nitrogen or oxygen and of a protein backbone or side-chain nitrogen or oxygen. Bridges were counted every 2 ns over the equilibrated window on the fully solvated trajectory, and the reported value is the mean number of such waters per frame. No angular criterion was applied, so the count is an upper bound on genuine bridging hydrogen bonds and is comparable between complexes rather than absolute.

Essential dynamics used principal component analysis, computed separately for each complex and not on a shared basis, of the Cα coordinates over the equilibrated window, sampled every 800 ps, after removal of translation and rotation by superposition on the first equilibrated frame. Projections onto the first two components are given in Figure 2.

### 4.8. MM-GBSA

Single-trajectory MM-GBSA effective interaction energies were computed with gmx_MMPBSA 1.6.5 [26], which wraps MMPBSA.py [27], using the generalised Born model igb = 5 with 0.15 M implicit salt and default non-polar surface-area term. Every fourth frame of the post-equilibration window (25–100 ns) was used, giving 94 frames per replicate, and each of the three replicates was analysed separately so that the reported spread is between replicates rather than between frames. Differences between compounds at a target are reported as Welch 95% confidence intervals on the difference in replicate means (n = 3 per group).

The receptor was defined as the protein plus its catalytic cofactor (NADP^+^ for DHFR, NAD^+^ for FabI, Zn^2+^ for LpxC; GyrB has none) and the ligand as the docked compound alone. Because the cofactor lines the binding site, assigning it to the ligand or omitting it changes the computed energy substantially. Water and counter-ions were stripped before analysis. No entropy term was computed; the values are therefore effective interaction energies and are not comparable to experimental binding free energies in absolute terms, only to each other within a target.

### 4.9. Molecular Graphics

Binding-mode panels (Figure 7) were rendered in PyMOL 3.1.0 [43]. A single representative frame was selected per complex by a rule fixed in advance and applied identically to every system: the replicate whose mean pocket retention is the median of the three, and within that replicate, the frame of the equilibrated window whose site-superposed ligand RMSD is closest to the median. Selecting the median rather than the best replicate and frame avoids presenting each compound at its most favourable, which would flatter the docking hits and the reference inhibitors alike. This is a representative frame, not a cluster centroid; no conformational clustering was performed, and the panels are illustrative of the ensemble averages in Table 3 rather than an independent structural analysis.

Polar partners are the residues with a donor or acceptor atom within 3.5 Å of a ligand donor or acceptor in that frame, counted with PyMOL’s polar-contact criterion. This is a single-frame geometric count and is therefore distinct from, and not expected to equal, the trajectory-averaged hydrogen-bond occupancies of Figure 6.

## 5. Conclusions

Across four druggability-validated *E. coli* antibacterial targets, docking scores placed three of four known inhibitors between the 21st and 48th percentile of an approved-drug library (triclosan, whose co-crystal pocket was used, ranked in the top 1%), while ranking large promiscuous compounds first, and neither pocket occupancy, contact-loss time, nor MM-GBSA rescoring over 3.6 μs of replicate molecular dynamics separated the two classes. Persistent protein–ligand hydrogen bonding did, at three of four targets (GyrB tentatively), by margins of 2–9-fold, with the reference inhibitor simultaneously the least mobile ligand. Interaction-specific analysis of short replicate simulations, benchmarked against a known active carried through the same pipeline, was therefore more informative in this small panel than either the docking score or gross pose stability, although confirmation requires multiple actives and property-matched decoys per target. Applying that benchmark to the hit list nominates three candidates, as hypotheses, for experimental testing: capmatinib at DHFR, metergoline at FabI and nilotinib at LpxC. Capmatinib ranks highest on these criteria, with pocket retention, pose stability and MM-GBSA energy showing no detectable difference from trimethoprim’s at n = 3 (no evidence of equivalence), while apparently binding through shape complementarity rather than the conserved diaminopyrimidine hydrogen-bond network. GyrB yielded no candidate. Selectivity over the human orthologue is the decisive next experiment. Enzyme-inhibition and whole-cell assays, together with assessment of outer-membrane permeability and efflux, are required before any antibacterial claim can be made.

## Figures and Tables

**Table 1:** Targets taken forward. All structures are *Escherichia coli*. Druggability is the fpocket score for the pocket enclosing the site anchor, computed with the catalytic cofactor retained.

Target	PDB	Resolution	Site Anchor	Cofactor Retained	Druggability	Native Redock RMSD
DHFR	1RX2 [22]	1.80 Å	folate	NADP^+^	0.96	0.67 Å
FabI	1QSG [23]	1.75 Å	triclosan	NAD^+^	0.94	0.50 Å
LpxC	3P3G [24]	1.65 Å	3P3 (LPC-009)	Zn^2+^	0.89	0.52 Å
GyrB	1KZN [25]	2.30 Å	clorobiocin	—	0.60	0.69 Å

**Table 2:** Docking rank of compounds with established activity at each target, within the 2070-compound approved-drug library.

Target	Reference	Affinity (kcal/mol)	Rank	Percentile
FabI	triclosan	−9.54	22/2067	1.0%
DHFR	methotrexate	−7.88	443/2068	21%
GyrB	novobiocin	−7.67	576/2069	28%
DHFR	trimethoprim	−7.43	762/2068	37%
DHFR	pyrimethamine	−7.14	988/2068	48%

**Table 3:** Post-equilibration (25–100 ns) means over three replicates. Reference inhibitors are shown in bold. In the contact-loss column, individual event times (ns) are listed first, followed by the number of replicates right-censored at 100 ns (initial contacts still above the 50% criterion); censored replicates are not included in any average.

Target	Compound	Role	H-Bonds	Ligand RMSD (Å)	Retention (%)	Contact-Loss Events (ns); Censored
DHFR	**trimethoprim**	**reference**	**3.6**	**1.29**	80.9 ± 8.1	none; 3/3 cens.
DHFR	capmatinib	hit	0.4	1.86	87.3 ± 6.8	none; 3/3 cens.
DHFR	lumacaftor	hit	0.4	4.14	84.8 ± 4.8	none; 3/3 cens.
LpxC	**LPC-009 (3P3)**	**reference**	**2.1**	**1.91**	79.0 ± 5.2	none; 3/3 cens.
LpxC	nilotinib	hit	0.8	4.71	65.8 ± 7.2	none; 3/3 cens.
LpxC	antrafenine	hit	0.5	7.04	56.2 ± 26.8	1; 2/3 cens.
GyrB	**novobiocin**	**reference**	**2.1**	**7.10**	52.7 ± 21.7	20, 10; 1/3 cens.
GyrB	entrectinib	hit	0.7	9.34	46.4 ± 3.9	40, 64, 41; 0/3 cens.
GyrB	lumacaftor	hit	1.0	7.61	43.9 ± 3.2	39, 23, 5; 0/3 cens.
FabI	triclosan	reference	0.4	5.59	50.4 ± 18.2	55, 75; 1/3 cens.
FabI	metergoline	hit	0.1	5.15	59.1 ± 10.2	19; 2/3 cens.
FabI	vibegron	hit	0.5	7.97	42.6 ± 11.1	38, 14; 1/3 cens.

**Table 4:** MM-GBSA effective interaction energies (kcal/mol; approximate estimates, entropy omitted), mean ± SD over three replicates. More negative is more favourable. By point estimate, the reference inhibitor is the most favourable compound at one of four targets.

Target	Compound	Role	ΔG_MM-GBSA_	Rank of 3
DHFR	capmatinib	hit	−35.2 ± 2.5	1
DHFR	lumacaftor	hit	−32.9 ± 3.1	3
DHFR	**trimethoprim**	**reference**	**−33.2 ± 0.7**	**2**
LpxC	**LPC-009 (3P3)**	**reference**	**−42.0 ± 7.2**	**1**
LpxC	nilotinib	hit	−32.8 ± 2.0	2
LpxC	antrafenine	hit	−28.8 ± 12.0	3
GyrB	entrectinib	hit	−43.2 ± 10.5	1
GyrB	**novobiocin**	**reference**	**−32.2 ± 11.9**	**2**
GyrB	lumacaftor	hit	−23.1 ± 2.3	3
FabI	metergoline	hit	−30.3 ± 4.7	1
FabI	triclosan	reference	−25.8 ± 1.5	2
FabI	vibegron	hit	−21.6 ± 3.1	3

**Table 5:** Candidate compounds, compared with the reference inhibitor of the same target. Values are post-equilibration (25–100 ns) means ± 1 SD over three replicates. Ligand efficiency is MM-GBSA per heavy atom.

Target	Compound	Retention (%)	Ligand RMSD (Å)	MM-GBSA (kcal/mol)	LE (kcal/mol per Heavy Atom)	H-Bonds
DHFR	**Capmatinib**	87.3 ± 6.8	1.86 ± 0.34	−35.2 ± 2.5	−1.13	0.4
DHFR	*Trimethoprim (ref.)*	80.9 ± 8.1	1.29 ± 0.20	−33.2 ± 0.7	−1.58	3.6
FabI	**Metergoline**	59.1 ± 10.2	5.15 ± 2.96	−30.3 ± 4.7	−1.01	0.1
FabI	*Triclosan (ref.)*	50.4 ± 18.2	5.59 ± 2.00	−25.8 ± 1.5	−1.52	0.4
LpxC	**Nilotinib**	65.8 ± 7.2	4.71 ± 2.75	−32.8 ± 2.0	−0.84	0.8
LpxC	*LPC-009 (3P3) (ref.)*	79.0 ± 5.2	1.91 ± 0.74	−42.0 ± 7.2	−1.24	2.1

## Data Availability

The original contributions presented in this study are included in the article/Supplementary Materials. Further inquiries can be directed to the corresponding author.

## Supplementary Materials

The following supporting information can be downloaded at: https://www.mdpi.com/article/10.3390/ijms27209017/s1.
