# Supplementary material for: Molecular Dynamics-Guided Triage of Approved Drugs Against Escherichia coli Resistance Targets Nominates Capmatinib, Metergoline and Nilotinib as Candidates for Experimental Testing
Source: Int J Mol Sci. 2026 Oct 12;27(20):9017. doi: 10.3390/ijms27209017 (PMC13705101; doi:10.3390/ijms27209017)
Supplement: Supplementary file 1 [file ijms-27-09017-s001.zip › ijms-4607335-supplementary.pdf]

Supplementary Materials

Molecular Dynamics-Guided Triage of Approved Drugs Against Escherichia coli Resistance Targets Nominates Capmatinib, Metergoline and Nilotinib as Candidates for Experimental Testing

Mena Abdelsayed [1,*,†] and Yassir Boulaamane [2,†]

[1] Lankenau Institute for Medical Research, Philadelphia, PA 19096, USA
[2] Laboratory of Innovative Technologies, National School of Applied Sciences of Tangier, Abdelmalek Essaadi University, Tetouan 93000, Morocco
* Correspondence: abdelsayedm@mlhs.org
† These authors contributed equally to this work.

Table S1. Pocket-druggability triage of all seven candidate structures

Druggability is the fpocket score for the pocket enclosing the site anchor, computed with catalytic cofactors retained. "Best anywhere" is the highest-scoring pocket found on the same structure, included to show that the low site scores are not an artefact of the whole protein scoring poorly.

Target	Organism	PDB	Site anchor	Cofactor kept	Site pocket	Distance to anchor (Å)	Druggability	Volume (Å³)	Best anywhere	Outcome
DHFR	*E. coli*	1RX2	FOL161	$NADP^+$	1	9.7	**0.960**	1927	0.960	advanced
FabI	*E. coli*	1QSG	TCL1302	NAD^+	1	0.8	**0.938**	591	0.938	advanced
LpxC	*E. coli*	3P3G	3P3401	Zn^{2+}	3	4.0	**0.885**	509	0.885	advanced
GyrB	*E. coli*	1KZN	CBN1	—	1	5.1	**0.596**	523	0.596	advanced
MurA	*E. coli*	1UAE	UD1420	—	17	3.2	**0.331**	1162	0.331	not pursued
TrxB	*E. coli*	1TDE	CYS135+CYS138	FAD	4	5.1	**0.154**	388	0.354	excluded
TrxB	*S. aureus*	4GCM	CYS134+CYS137	FAD	14	7.5	**0.008**	370	0.886	excluded

The cofactor-stripping artefact quantified: *E. coli* thioredoxin reductase (1TDE) scores **0.154** at its catalytic cysteine site with FAD retained, but **0.993** when all HETATM records are removed, on a pocket centred 4.0 Å from the FAD that was deleted. Retaining cofactors is therefore not a detail of preparation, but a determinant of which targets a triage selects.

Table S2. Simulation system composition

Dodecahedral boxes with a 1.0 nm solute–boundary margin, TIP3P water, neutralised with Na^+ counter-ions.

System	Target	Compound	Atoms	Box edge (nm)	Waters	Na^+

DB13520_FabI	FabI	Metergoline	40,062	8.32	12,032	5
DB14895_FabI	FabI	Vibegron	40,067	8.32	12,033	5
DB08604_FabI	FabI	Triclosan	40,057	8.32	12,042	5
DB04868_LpxC	LpxC	Nilotinib	40,731	8.35	11,990	5
DB01419_LpxC	LpxC	Antrafenine	40,729	8.35	11,987	5
3P3_LpxC	LpxC	LpxC inhibitor (3P3)	40,766	8.35	12,007	5
DB11791_DHFR	DHFR	Capmatinib	23,877	7.00	7,084	15
DB09280_DHFR	DHFR	Lumacaftor	23,871	7.00	7,081	15
DB00440_DHFR	DHFR	Trimethoprim	23,874	7.00	7,086	15
DB11986_GyrB	GyrB	Entrectinib	27,941	7.40	8,237	11
DB09280_GyrB	GyrB	Lumacaftor	27,944	7.40	8,246	11
DB01051_GyrB	GyrB	Novobiocin	27,940	7.40	8,235	11

Table S3. MM-GBSA energy decomposition

Mean over three replicates, kcal/mol. ΔE_{vdW} and ΔE_{elec} are the gas-phase molecular-mechanics terms; ΔG_{GB} and ΔG_{SA} are the polar and non-polar solvation terms. No entropy term was computed, so values are effective interaction energies comparable within a target but not to experiment.

**Target**	**Compound**	**Role**	ΔE_{vdW}	ΔE_{elec}	ΔG_{GB}	ΔG_{SA}	ΔG_{total}
FabI	Metergoline	hit	-39.9	-9.5	24.1	-4.9	-30.3
FabI	Vibegron	hit	-33.6	-14.9	31.3	-4.4	-21.6
FabI	**Triclosan**	reference	-30.5	-5.4	13.9	-3.8	**-25.8**
LpxC	Nilotinib	hit	-49.7	-16.5	40.0	-6.6	-32.8
LpxC	Antrafenine	hit	-39.5	-12.4	28.7	-5.5	-28.8
LpxC	**LpxC inhibitor (3P3)**	reference	-38.1	-71.5	74.2	-6.5	**-42.0**
DHFR	Capmatinib	hit	-45.3	-20.0	35.9	-5.6	-35.2
DHFR	Lumacaftor	hit	-43.3	-16.3	32.6	-5.9	-32.9
DHFR	**Trimethoprim**	reference	-36.7	-19.2	27.6	-4.9	**-33.2**
GyrB	Entrectinib	hit	-53.8	-21.2	38.2	-6.5	-43.2
GyrB	Lumacaftor	hit	-32.1	-8.0	21.4	-4.4	-23.1
GyrB	**Novobiocin**	reference	-46.9	-19.8	40.6	-6.1	**-32.2**

At FabI, DHFR and GyrB, the compound with the most favourable ΔE_{vdW} is also the one with the most favourable ΔG_{total}, so the ranking follows buried contact area. LpxC is the exception: LPC-009 has a *smaller* van der Waals term than nilotinib (−38.1 versus −49.7) yet wins on ΔE_{elec} (−71.5 versus −16.5), consistent with hydroxamate chelation of the catalytic Zn^{2+}. This term is largely offset by polar solvation (ΔEelec + ΔGGB = +2.7 for LPC-009 versus +23.5 for nilotinib), so it is the net polar balance, not ΔEelec alone, that offsets nilotinib's van der Waals advantage.

Differences in ΔGtotal, hit minus reference, kcal/mol (negative favours the hit), with Welch 95% confidence intervals computed from the replicate means and SDs (n = 3 per group): FabI, metergoline minus triclosan −4.5 (−15.0 to +6.0) and vibegron minus triclosan +4.2 (−2.3 to +10.7); LpxC, nilotinib minus LPC-009 +9.2 (−7.2 to +25.6) and antrafenine minus LPC-009 +13.2 (−11.3 to +37.7); DHFR, capmatinib minus trimethoprim −2.0 (−7.7 to +3.7) and lumacaftor minus trimethoprim +0.3 (−6.9 to +7.5); and GyrB, entrectinib minus novobiocin −11.0 (−36.6 to +14.6) and lumacaftor minus novobiocin +9.1 (−19.1 to +37.3). Every interval includes zero, so these data establish neither a ranking nor equivalence.

Table S4. Per-replicate values underlying the main-text means

Post-equilibration window (25–100 ns). Each row is one complex; the three values are the three independent replicates.

Target	Compound	Role	Pocket retention (%)	Ligand RMSD (Å)	H-bonds	Contact-loss time (ns)
FabI	Metergoline	hit	69, 60, 49	2.62, 4.43, 8.41	0.1, 0.2, 0.1	100*, 100*, 19
FabI	Vibegron	hit	54, 32, 41	6.08, 7.40, 10.42	0.5, 0.4, 0.6	100*, 38, 14
FabI	**Triclosan**	reference	43, 71, 37	6.04, 3.41, 7.33	0.2, 0.7, 0.4	55, 100*, 75
LpxC	Nilotinib	hit	74, 61, 62	2.96, 7.88, 3.30	1.0, 0.5, 0.9	100*, 100*, 100*
LpxC	Antrafenine	hit	30, 55, 84	10.01, 7.75, 3.38	0.0, 0.6, 1.0	1, 100*, 100*
LpxC	**LpxC inhibitor (3P3)**	reference	79, 74, 84	1.33, 2.74, 1.65	2.1, 2.3, 1.8	100*, 100*, 100*
DHFR	Capmatinib	hit	80, 91, 91	1.85, 1.53, 2.20	0.4, 0.4, 0.3	100*, 100*, 100*
DHFR	Lumacaftor	hit	82, 82, 90	5.00, 2.96, 4.46	0.2, 0.3, 0.6	100*, 100*, 100*
DHFR	**Trimethoprim**	reference	79, 90, 74	1.18, 1.17, 1.52	3.5, 3.6, 3.6	100*, 100*, 100*
GyrB	Entrectinib	hit	51, 44, 44	9.72, 8.78, 9.53	1.1, 0.5, 0.5	40, 64, 41
GyrB	Lumacaftor	hit	43, 47, 41	7.09, 7.35, 8.40	0.8, 0.4, 1.7	39, 23, 5
GyrB	**Novobiocin**	reference	77, 45, 36	4.15, 7.57, 9.57	2.8, 1.5, 2.1	100*, 20, 10

* Initial contacts still above the 50% criterion at 100 ns (right-censored). Every value at 100 ns is censored.

Table S5. Convergence of each metric over the sampling window

Mean over the first half (25–62.5 ns) versus the second half (62.5–100 ns) of the post-equilibration window, pooled over three replicates. Δ is second minus first.

Target	Compound	Retention 1st/2nd/Δ	Ligand RMSD 1st/2nd/Δ	H-bonds 1st /2nd/Δ
FabI	Metergoline	58/60/+2	5.33/4.97/-0.36	0.2/0.1/-0.1
FabI	Vibegron	45/40/-6	7.27/8.67/+1.39	0.4/0.6/+0.2
FabI	**Triclosan**	56/44/-12	5.27/5.92/+0.65	0.6/0.3/-0.3
LpxC	Nilotinib	67/64/-3	4.40/5.03/+0.63	0.8/0.7/-0.1
LpxC	Antrafenine	58/55/-3	7.21/6.88/-0.33	0.5/0.6/+0.0
LpxC	**LpxC inhibitor (3P3)**	79/79/+1	1.86/1.96/+0.11	2.2/2.0/-0.2
DHFR	Capmatinib	88/87/-0	1.90/1.82/-0.07	0.4/0.4/+0.1

DHFR	Lumacaftor	85/84/-1	3.99/4.29/+0.30	0.4/0.3/-0.1
DHFR	**Trimethoprim**	81/81/+1	1.34/1.24/-0.09	3.6/3.6/-0.0
GyrB	Entrectinib	50/43/-8	8.96/9.72/+0.75	0.9/0.5/-0.5
GyrB	Lumacaftor	44/44/+1	6.96/8.27/+1.32	1.2/0.8/-0.4
GyrB	**Novobiocin**	53/53/+0	6.36/7.83/+1.47	2.1/2.2/+0.1

Across all twelve complexes, the largest drift between halves is 12 percentage points in pocket retention, 1.47 Å in ligand RMSD and 0.5 in hydrogen-bond count. The three largest drifts all occur in complexes whose ligand is leaving the site during the trajectory (triclosan at FabI, entrectinib and novobiocin at GyrB), where a difference between halves reflects the dissociation being measured rather than insufficient sampling. At DHFR and LpxC, where all but one replicate kept its initial contacts, retention agrees between halves to within 3 percentage points. The discrimination reported in the main text does not depend on which half of the window is analysed. Agreement between the two halves is, however, limited evidence of convergence.

Table S6. Contact occupancy, contact specificity and water bridges

Mean ± SD over three replicates, post-equilibration window. *Contacted* is the number of residues within 4.0 Å of the ligand in more than 5% of frames; *core* is the subset held in at least 80% of frames; *specificity* is their ratio. *Water bridges* is the mean number of waters simultaneously within 3.5 Å of a ligand and a protein polar atom.

Target	Compound	Role	Contacted	Core	Specificity	Water bridges
FabI	Metergoline	hit	22 ± 2	11 ± 4	0.51 ± 0.19	0.7 ± 0.4
FabI	Vibegron	hit	26 ± 1	5 ± 0	0.20 ± 0.01	1.8 ± 0.3
FabI	**Triclosan**	reference	21 ± 4	6 ± 4	0.29 ± 0.23	**0.6 ± 0.2**
LpxC	Nilotinib	hit	31 ± 4	15 ± 1	0.47 ± 0.07	2.9 ± 0.6
LpxC	Antrafenine	hit	29 ± 7	13 ± 7	0.45 ± 0.14	1.4 ± 0.7
LpxC	**LpxC inhibitor (3P3)**	reference	27 ± 4	17 ± 3	0.66 ± 0.20	**5.2 ± 0.2**
DHFR	Capmatinib	hit	21 ± 0	13 ± 1	0.63 ± 0.03	2.0 ± 0.2
DHFR	Lumacaftor	hit	22 ± 2	13 ± 1	0.58 ± 0.07	2.1 ± 0.7
DHFR	**Trimethoprim**	reference	19 ± 1	13 ± 1	0.72 ± 0.06	**2.9 ± 0.1**
GyrB	Entrectinib	hit	35 ± 4	15 ± 4	0.43 ± 0.10	2.4 ± 0.6
GyrB	Lumacaftor	hit	28 ± 2	8 ± 2	0.28 ± 0.07	2.5 ± 0.2
GyrB	**Novobiocin**	reference	34 ± 6	12 ± 5	0.35 ± 0.17	**5.7 ± 0.1**

Contact specificity favours the reference inhibitor at DHFR (0.72) and LpxC (0.66) but not at GyrB or FabI, so it discriminates at two of four targets, less reliably than either hydrogen bonds or water bridges, which both discriminate at three. It is reported for completeness rather than as a recommended descriptor.

Table S7. Per-residue hydrogen-bond occupancy

Fraction of equilibrated frames in which a given protein residue forms at least one direct hydrogen bond to the ligand (same criterion as Section 4.6). *Partners* is the mean number of residues engaged at all; *peak* is the highest single-residue occupancy; *persistent* counts residues at or above 50% in all three replicates.

Target	Compound	Role	Partners	Peak occupancy	Persistent (all 3 reps)
FabI	Metergoline	hit	1.7	0.06	0
FabI	Vibegron	hit	6.3	0.26	0
FabI	**Triclosan**	reference	3.3	**0.31**	0
LpxC	Nilotinib	hit	3.3	0.39	0
LpxC	Antrafenine	hit	2.0	0.43	0
LpxC	**LpxC inhibitor (3P3)**	reference	6.0	**0.65**	0
DHFR	Capmatinib	hit	4.3	0.21	0
DHFR	Lumacaftor	hit	5.3	0.10	0
DHFR	**Trimethoprim**	reference	5.0	**0.99**	3
GyrB	Entrectinib	hit	6.7	0.27	0
GyrB	Lumacaftor	hit	4.7	0.34	0
GyrB	**Novobiocin**	reference	13.3	**0.31**	0

Only trimethoprim shows residue-specific engagement by this measure, holding Ile5, Ile94 and Asp27 at 0.95–0.99 occupancy in all three replicates. The LpxC reference reaches 0.65 at one residue. Novobiocin forms a comparable *number* of hydrogen bonds to trimethoprim (2.1 versus 3.6, Figure 6) but spreads them over 13.3 residues with a peak occupancy of 0.31, i.e., it redistributes its polar contacts instead of holding a defined set.

Supplementary Figures

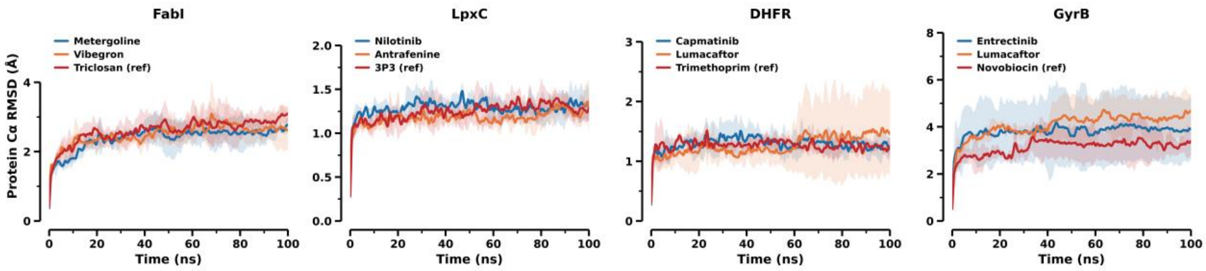

Figure S1. Protein Cα RMSD against the energy-minimised starting structure, grouped by target. Lines are the mean of three replicates, shaded bands ± 1 SD (sample SD, n = 3); mean traces carry a 1 ns running mean for legibility. GyrB shows the highest RMSD (3.3–4.3 Å), localised to the rebuilt 101–115 loop.

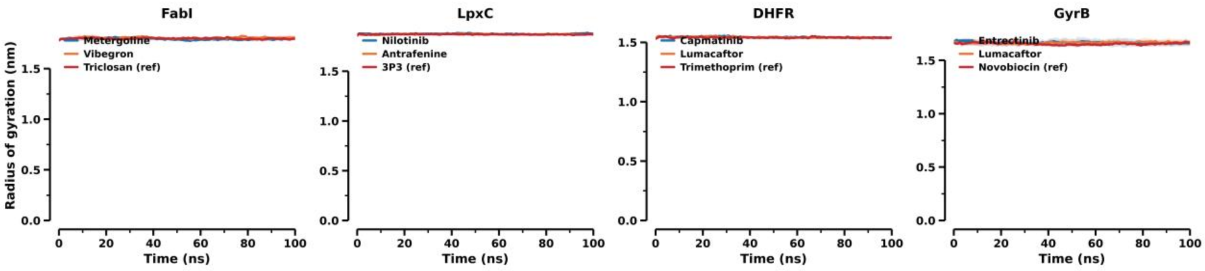

Figure S2. Radius of gyration. All twelve complexes are constant over 25–100 ns, including GyrB at 1.64–1.70 nm, confirming that no fold unfolded and that the rebuilt GyrB chain does not separate under periodic boundary conditions.

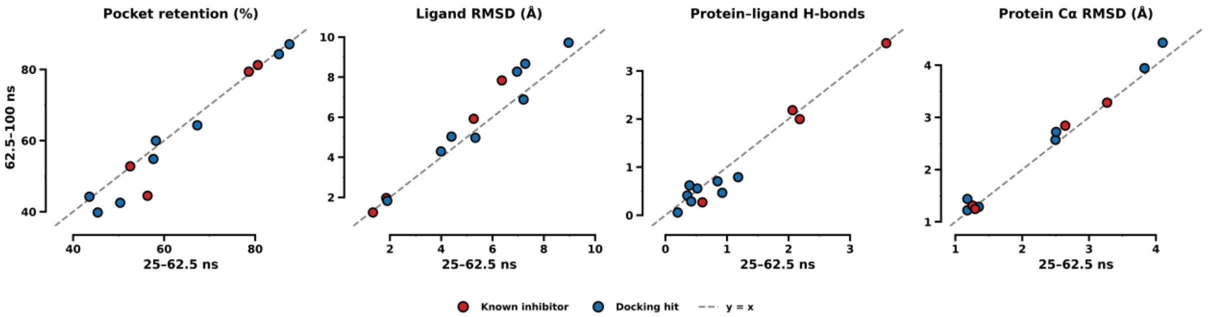

Figure S3. Convergence of each metric: mean over the first half of the sampling window (25–62.5 ns, abscissa) against the second half (62.5–100 ns, ordinate), one point per complex. The dashed line is y = x. Points lie close to it for every metric; the largest departures are the complexes in which the ligand is dissociating during the trajectory. Agreement between the two halves is limited evidence of convergence.

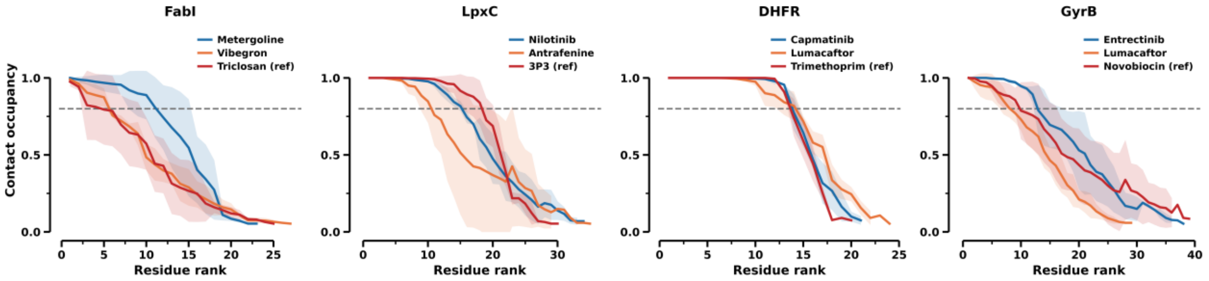

Figure S4. Per-residue contact occupancy, sorted in descending order, one curve per complex (mean of three replicates, band ± 1 SD). The dashed line marks the 0.80 threshold used to define a core contact. A steep curve with many residues above the line indicates a ligand holding a defined set of residues; a shallow curve indicates transient grazing contacts.

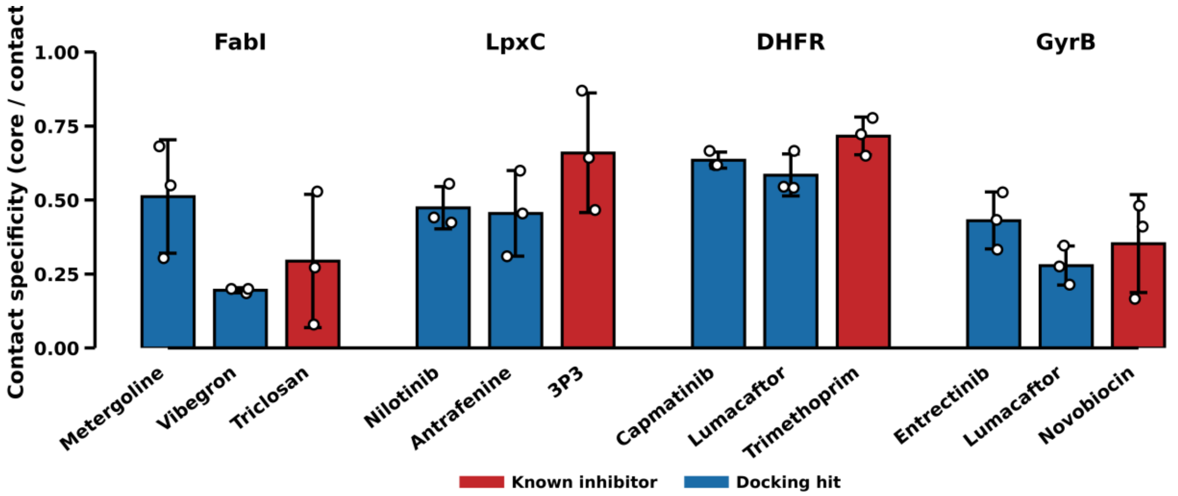

Figure S5. Contact specificity, the ratio of core contacts to all residues contacted in more than 5% of frames. The known inhibitor ranks highest at DHFR and LpxC only, so this descriptor discriminates at two of four targets.

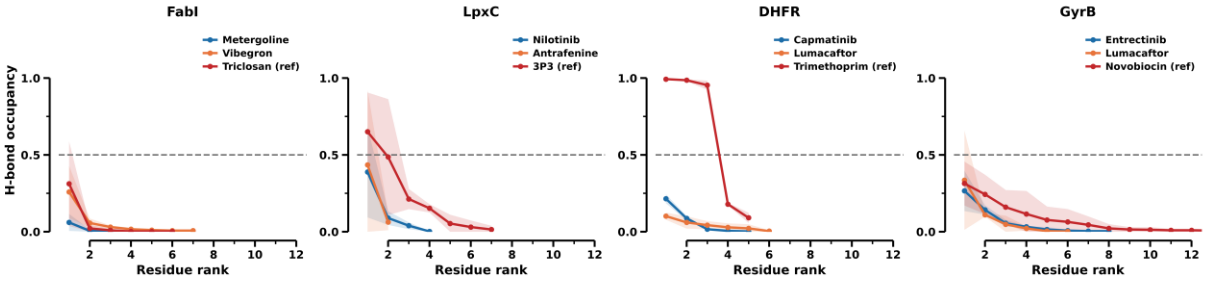

Figure S6. Per-residue hydrogen-bond occupancy, sorted in descending order, one curve per complex (mean of three replicates, band ± 1 SD). The dashed line marks 50% occupancy. Only trimethoprim rises above it for more than one residue; the other references form as many hydrogen bonds in total but distribute them among transient partners.
